# *Escherichia coli-* and *Staphylococcus aureus*-induced mastitis differentially modulate transcriptional responses in neighbouring uninfected bovine mammary gland quarters

**DOI:** 10.1186/1471-2164-14-36

**Published:** 2013-01-16

**Authors:** Kirsty Jensen, Juliane Günther, Richard Talbot, Wolfram Petzl, Holm Zerbe, Hans-Joachim Schuberth, Hans-Martin Seyfert, Elizabeth J Glass

**Affiliations:** 1 Division of Infection & Immunity, The Roslin Institute and Royal (Dick) School of Veterinary Studies, University of Edinburgh, Easter Bush, Midlothian EH25 9RG, UK; 2 Leibniz Institute for Farm Animal Biology, Dummerstorf, Germany; 3 ARK-Genomics Facility, The Roslin Institute and R(D)SVS, Easter Bush, Midlothian, Edinburgh, EH25 9RG, UK; 4 Clinic for Ruminants, Ludwig-Maximilians-University, Munich, Germany; 5 Institute of Immunology, University of Veterinary Medicine, Hannover, Germany

**Keywords:** Bovine, Mastitis, *Escherichia coli*, *Staphylococcus aureus*, Microarray

## Abstract

**Background:**

The most important disease of dairy cattle is mastitis, caused by the infection of the mammary gland by various micro-organisms. Although the transcriptional response of bovine mammary gland cells to *in vitro* infection has been studied, the interplay and consequences of these responses in the *in vivo* environment of the mammary gland are less clear. Previously mammary gland quarters were considered to be unaffected by events occurring in neighbouring quarters. More recently infection of individual quarters with mastitis causing pathogens, especially *Escherichia coli*, has been shown to influence the physiology of neighbouring uninfected quarters. Therefore, the transcriptional responses of uninfected mammary gland quarters adjacent to quarters infected with two major mastitis causing pathogens, *E. coli* and *Staphylococcus aureus*, were compared.

**Results:**

The bacteriologically sterile, within-animal control quarters exhibited a transcriptional response to the infection of neighbouring quarters. The greatest response was associated with *E. coli* infection, while a weaker, yet significant, response occurred during *S. aureus* infection. The transcriptional responses of these uninfected quarters included the enhanced expression of many genes previously associated with mammary gland infections. Comparison of the transcriptional response of uninfected quarters to *S. aureus* and *E. coli* infection identified 187 differentially expressed genes, which were particularly associated with cellular responses, e.g. response to stress. The most affected network identified by Ingenuity Pathway analysis has the immunosuppressor transforming growth factor beta 1 (TGFB1) at its hub and largely consists of genes more highly expressed in control quarters from *S. aureus* infected cows.

**Conclusions:**

Uninfected mammary gland quarters reacted to the infection of neighbouring quarters and the responses were dependent on pathogen type. Therefore, bovine udder quarters exhibit interdependence and should not be considered as separate functional entities. This suggests that mastitis pathogens not only interact directly with host mammary cells, but also influence discrete sites some distance away, which will affect their response to the subsequent spread of the infection. Understanding the underlying mechanisms may provide further clues for ways to control mammary gland infections. These results also have implications for the design of experimental studies investigating immune regulatory mechanisms in the bovine mammary gland.

## Background

Mastitis, the inflammation of the mammary gland, is the most important disease of dairy cattle, with respect to the frequency of occurrence, animal welfare and economic cost, which is estimated to approach $2 billion annually in the US alone (reviewed by [1]). Mastitis is most frequently caused by infection by a variety of bacteria, of which *Staphylococcus aureus* and *Escherichia coli* are amongst the most important gram-positive and gram-negative bacteria respectively (reviewed by [2]). There is considerable variation in the mastitic disease caused by these two pathogens. *E. coli* intramammary infections often result in acute mastitis with severe clinical manifestations, which usually resolves within a few days. In contrast, symptoms induced by *S. aureus* infection are usually less severe, even asymptomatic, and the infection can persist for long periods of time.

Current control strategies largely involve hygiene practices on the farm and the direct administration of antibiotics into udder quarters exhibiting clinical signs of infection. However, many infections are asymptomatic and there is a desire to minimize the use of antibiotics. Therefore, there is a need to develop new and improved control strategies. To achieve this goal our understanding of events that occur during infection must be improved. The use of global transcriptomic studies, either involving the use of microarrays or more recently Next Generation Sequencing, is ideally suited to investigate the complex host-pathogen interplay occurring during mastitis, by simultaneously quantifying the expression of thousands of genes. Several studies have used microarrays to investigate the bovine mammary gland response to infection with particular bacteria, including *E. coli*[3,4], *Streptococcus uberis*[5,6] and *S. aureus*[7]. A direct comparison of the induced transcriptional responses identified in these studies is difficult because of differences in the experimental design and the microarray platforms used. However, meta-analyses of such transcriptional studies have recently been reported [8,9]. The meta-analysis reported by Genini et al. [8] identified a common transcriptional response in the mammary glands of several species to different pathogens and identified several new pathways not previously identified in the individual studies. However, a considerable amount of information was excluded from this meta-analysis because of the limited overlap of probes included on the microarrays used in the original studies. Therefore, we designed an infection study to investigate and compare the early transcriptional response of the bovine mammary gland to challenge with *S. aureus* and *E. coli* using a single microarray platform.

The experimental model used in this study involved the sequential inoculation of udder quarters with bacteria over the course of the study period, leaving one quarter uninfected as a within animal control [10]. The use of within animal controls is a common and accepted practice because the udder quarters are generally considered to be separate, independent anatomical structures [3-7,11-16] and reduces the between animal variation seen in outbred species such as cattle, as well as addressing the 3Rs, Reduction, Replacement and Refinement, the principles behind the ethical framework for conducting animal experiments. However, analysis of the microarray experiment reported here revealed that *E. coli* infection of mammary gland quarters can have a profound effect on gene expression in other, uninfected quarters. This conclusion is supported by another transcriptional study comparing uninfected quarters from healthy and *E. coli* infected cattle [17]. Furthermore, analysis of the microarray data presented here suggests that a similar, but distinct, effect also occurs during *S. aureus* infection.

## Results

### Microarray analysis of the transcriptional response of the bovine mammary gland to *E. coli* and *S. aureus* infection

A strictly defined model for *E. coli*- and *S. aureus*-induced mastitis was developed involving the sequential infection of three udder quarters whilst the fourth quarter acted as a control [10]. RNA samples collected from each quarter of the twelve animals included in this study were interrogated using the ARK-Genomics *B. taurus* 20 K cDNA microarray. Initially, the intramammary transcriptional response to *E. coli* and *S. aureus* infection was investigated by comparing the expression of genes in infected quarters with those expressed in the uninfected, within animal control quarters. Only five clones exhibited differential expression during *S. aureus* infection (false discovery rate (*FDR)* ≤ 0.05, fold difference ≥ 2) (Table 1). The clone AJ813772, which is currently unannotated and only matches EST sequences and an intergenic region of bovine chromosome 6, was significantly down-regulated 2.6 fold at 6 hours post infection. Similarly, two of the four clones that exhibit differential expression at 12 hours post infection are currently unannotated. These clones, AJ819694 and BF775518, were up-regulated 2.2 and 3.7 fold respectively. The other clones match lipopolysaccharide binding protein (LBP) and superoxide dismutase 2 (SOD2), which were up-regulated 4.8 and 5.9 fold respectively. No clones were identified as being differentially expressed at 24 or 72 hours post infection with *S. aureus*.

**Table 1:** **Number of genes identified as being differentially expressed (*FDR*** **≤ 0.05, fold change ≥ 2) during *Escherichia coli *and *Staphylococcus aureus *infection by comparing samples to respective within-animal control samples**

**Hours post infection**	***E. coli***	***S. aureus***
6	0	1
12	6	4
24	1048	0
72	-	0

The *E. coli* infection induced clinical symptoms in cattle within 12 hours of infection, including; increased somatic cell count (SCC) in milk from infected quarters, decreased milk production, fever and decreased blood leukocyte counts [10]. It was, therefore, surprising that large numbers of differentially expressed genes were only detected in quarters infected for 24 hours (Table 1). No clones exhibited differential expression at 6 hours post infection, whilst only six were differentially expressed at 12 hours post infection (*FDR* ≤ 0.05, fold difference ≥ 2). The currently unannotated clone C0882029 was down-regulated 2.1 fold. The remaining five clones were up-regulated and represent; chemokine (C-C motif) ligand 2 (3.9 fold), R3H domain containing 1 (3.3 fold), BTG family, member 2 (3.0 fold), Tribbles homolog 1 (2.4 fold) and chromosome 17 open reading frame 42 (2.3 fold). In contrast, 1048 clones were differentially expressed at 24 hours post infection. The two most up-regulated clones represent S100A8 and S100A12, which exhibited 21.9 and 19.8 fold up-regulation respectively. The two most down-regulated clones represent lipin 1 and WD repeat domain, phosphoinositide interacting 1 (WIPI1), which exhibited 5.4 and 5.2 fold down-regulation respectively.

### Preliminary comparison of control quarters

**Figure 1:**
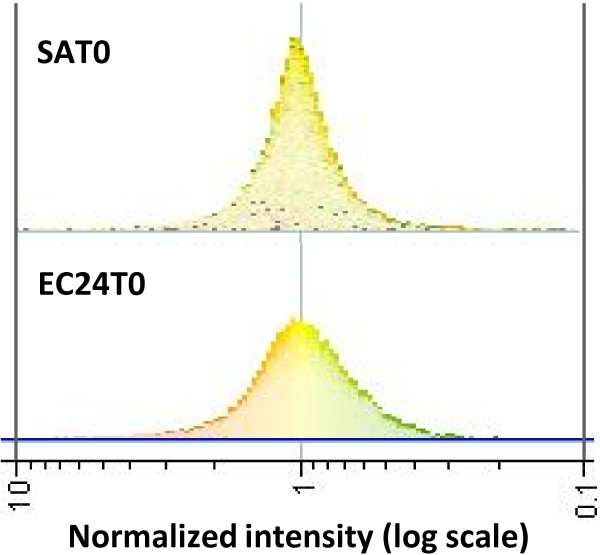
**Differential expression in uninfected udder quarters from animals infected with *Escherichia coli *and *Staphylococcus aureus. ***Preliminary analysis of the microarray data revealed different patterns of gene expression in control quarters from animals infected with *Escherichia coli *and *Staphylococcus aureus*. The log normalized intensity values (sample [Cy5]/reference [Cy3]) were plotted for the combined data from control quarters from animals infected with *E. coli *(EC24T0) and *S. aureus *(SAT0) using GeneSpring (Agilent). Both plots have a normal-type distribution around 1. EC24T0 have a broader peak of intensity values suggesting a greater range of expression compared to SAT0.

The low numbers of differentially expressed genes (DEG) detected in infected udder quarters, especially those infected with *E. coli*, were unexpected. Recently over 70 genes were found to be differentially expressed in the bovine mammary gland 12 hours after *E. coli* infection [3]. Furthermore, 183 genes were found to be differentially expressed 16 hours post *S. aureus* infection of the bovine mammary gland [7] using the smaller bovine innate immune microarray [18]. The discrepancy between these results may relate to the strain of bacterium used or the tissue collection procedure. Another potential explanation is that the low number of DEG may be due to the base-line provided by the control quarters. To investigate this possibility the transcriptomes of the *E. coli* control quarters (EC24T0) and control quarters from both *S. aureus* infection experiments (SAT0) were compared. Preliminary analysis using GeneSpring (Agilent) highlighted differences between the transcription profiles. Log intensity plots (sample/reference) of EC24T0 and SAT0 had different shapes (Figure 1). Whilst both had normal type distributions centred around one, EC24T0 had a broader distribution than SAT0. This suggests that the transcription profile of SAT0 is more similar to the reference sample, a mixed pool of all the RNA samples, than EC24T0.

### Genes exhibiting differential expression in control quarters from animals infected with *E. coli* and *S. aureus*

Due to the observed difference in the transcription profiles of RNA from the control quarters of *E. coli* and *S. aureus* infected animals, the transcriptomes of these samples were compared. Only the control quarters from the four animals infected with *S. aureus* for up to 24 hours (SA24T0) were included in this comparison. The analysis revealed that 255 clones exhibited significantly different expression levels in the control quarters (*FDR* ≤ 0.05, fold difference ≥ 2). The 255 clones, of which 212 are currently annotated, represent 187 genes and are listed in Additional file 1. Therefore, the uninfected quarters, which remained bacteriologically negative [10], exhibited significantly different transcriptional profiles, which were dependent on whether the animals had been infected in neighbouring quarters with *E. coli* or *S. aureus*. Ninety nine clones, representing 77 genes, were expressed at significantly higher levels in SA24T0 than in EC24T0. The clone AJ814393, which exhibited the highest expression level compared to EC24T0, a 4.6 fold difference, is currently unannotated, matching only EST sequences and an intergenic region of bovine chromosome 14. The remaining 156 clones, representing 110 genes, were expressed at significantly higher levels in EC24T0 than in SA24T0. The clone exhibiting the highest expression level compared to SA24T0, with a 6.6 fold difference, represents FK506 binding protein 5 (FKBP5).

The DEG list was examined using the web-based Database of Annotation, Visualization and Integrated Discovery (DAVID) [19,20]. Human RefSeq accession numbers were used in the analysis due to the limitations in the current bovine annotation. Gene Ontology (GO) Biological Process classifications were assigned to 144 (77%) of the DEG. Gene enrichment analysis was carried out using the default human background and the probability of each functional category was calculated using the modified Fisher Extract test. The two GO terms exhibiting the greatest enrichment were; Cellular Compartment Organization and Biogenesis and Response to Stress (Table 2). Other enriched GO terms included Cell Death and Cell Activation (Table 2). These results indicate that a large cellular response to the infection of neighbouring quarters was occurring in EC24T0 and/or SA24T0.

**Table 2:** **Major gene ontology biological process classifications represented by genes differentially expressed between control quarters from cows infected with *E. coli *(EC24T0) and *S. aureus *(SA24T0)**

**Clone accession No.**	**Gene symbol**	**Gene**	**Fold difference**
**CELLULAR COMPONENT ORGANIZATION AND BIOGENESIS**			
**EC24T0 > SA24T0**			
AM017607	ARPC5L	actin related protein 2/3 complex, subunit 5-like	2.52
CN821828	AGT	angiotensinogen	3.73
AM034974	BZW2	basic leucine zipper and W2 domains 2	2.48
BG693016	BTG1	B-cell translocation gene 1, anti-proliferative	2.67
BF076579	BAX	BCL2-associated X protein	2.02
AJ818096	CEBPG	CCAAT/enhancer binding protein (C/EBP), gamma	2.19
CO888526	EMP1	epithelial membrane protein 1	2.30
CN824732	EIF1	eukaryotic translation initiation factor 1	2.08
CO894798	EIF1B	eukaryotic translation initiation factor 1B	2.03
CO888678	EIF3D	eukaryotic translation initiation factor 3, subunit D	2.03
AJ691100	EIF4A2	eukaryotic translation initiation factor 4A, isoform 2	2.52
CN823782	GABARAPL1	GABA(A) receptor-associated protein like 1	3.10
AJ818246	HSPD1	heat shock 60 kDa protein 1	2.34
AJ673268	HIST1H2AC	histone cluster 1, H2ac	3.30
CO884622	MID1IP1	MID1 interacting protein 1	2.18
AJ819252	MKI67IP	MKI67 (FHA domain) interacting nucleolar phosphoprotein	2.29
AJ673531	NDP	Norrie disease	3.44
AM037100	NUPR1	nuclear protein 1	2.27
CO888501	PDPK1	3-phosphoinositide dependent protein kinase-1	2.45
AM030492	PPP1R9B	protein phosphatase 1, regulatory (inhibitor) subunit 9B	2.63
CO890613	RAB43	RAB43, member RAS oncogene family	2.29
AJ813402	SETD7	SET domain containing (lysine methyltransferase) 7	2.40
AJ813146	SNRPG	small nuclear ribonucleoprotein polypeptide G	2.73
CO886482	SLC3A2	solute carrier family 3 (activators of dibasic and neutral amino acid transport), member 2	2.54
CO892251	SLC7A8	solute carrier family 7 (cationic amino acid transporter, y + system), member 8	4.39
CO890843	STOM	stomatin	3.65
CO880484	SNTA1	syntrophin, alpha 1	4.49
CO887666	UTP14A	UTP14, U3 small nucleolar ribonucleoprotein, homolog A	2.26
**SA24T0 > EC24T0**			
CO889600	AMOT	angiomotin	2.12
CO889378	CNN1	calponin 1, basic, smooth muscle	2.49
CN822971	CALR	calreticulin	2.36
BF604066	CD74	CD74 molecule, major histocompatibility complex, class II invariant chain	2.43
CO888048	CXCL12	chemokine (C-X-C motif) ligand 12	3.23
CO202183	FEZ1	fasciculation and elongation protein zeta 1	3.13
CO873026	FLNA	filamin A, alpha	2.31
AJ692384	GLMN	glomulin, FKBP associated protein	2.01
CN824202	GSK3B	glycogen synthase kinase 3 beta	2.02
CO887046	LRRC4C	leucine rich repeat containing 4C	2.92
CO872154	LASP1	LIM and SH3 protein 1	2.09
AM025652	MRPL32	mitochondrial ribosomal protein L32	2.06
CO893984	MYO9B	myosin IXB	4.03
CO889939	MYH11	myosin, heavy chain 11, smooth muscle	2.70
AM018723	PIGR	polymeric immunoglobulin receptor	2.03
AJ687977	SEC24D	SEC24 related gene family, member D	2.02
CO894656	SENP2	SUMO1/sentrin/SMT3 specific peptidase 2	3.32
CO876411	THY1	Thy-1 cell surface antigen	2.10
pTGFB1*	TGFB1	transforming growth factor beta 1	2.32
AJ814326	B4GALT1	UDP-Gal:betaGlcNAc beta 1,4- galactosyltransferase polypeptide 1	2.45
AM033343	VIM	vimentin	2.36
CO880794	WIPF1	WAS/WASL interacting protein family, member 1	2.34
**RESPONSE TO STRESS**			
**EC24T0 > SA24T0**			
AM006216	ATF4	activating transcription factor 4	2.07
CN821828	AGT	angiotensinogen	3.73
BF076579	BAX	BCL2-associated X protein	2.02
AJ818096	CEBPG	CCAAT/enhancer binding protein (C/EBP), gamma	2.19
BF606419	CXCL2	chemokine (C-X-C motif) ligand 2	2.03
CO889145	CSDA	cold shock domain protein A	3.16
CN824732	EIF1	eukaryotic translation initiation factor 1	2.08
CO892245	GADD45G	growth arrest and DNA-damage-inducible, gamma	2.91
AJ818246	HSPD1	heat shock 60 kDa protein 1	2.34
CN824191	HSP90AB1	heat shock protein 90 kDa alpha (cytosolic), class B member 1	2.25
AJ818172	HIF1A	hypoxia-inducible factor 1, alpha subunit	2.41
AM013900	LBP	lipopolysaccharide binding protein	2.79
CO888501	PDPK1	3-phosphoinositide dependent protein kinase-1	2.45
CN823413	SEPP1	selenoprotein P, plasma, 1	2.12
CO886480	STAT3	signal transducer and activator of transcription 3	2.25
AM025615	TRIB3	tribbles homolog 3	3.02
**SA24T0 > EC24T0**			
CO888048	CXCL12	chemokine (C-X-C motif) ligand 12	3.23
CO883340	CFD	complement factor D	2.31
CN824202	GSK3B	glycogen synthase kinase 3 beta	2.02
AJ820250	IDH1	isocitrate dehydrogenase 1 (NADP+), soluble	3.36
CO886661	RECQL5	RecQ protein-like 5	2.60
CO887968	TYMS	thymidylate synthetase	3.35
AJ695939	TFPI2	tissue factor pathway inhibitor 2	2.03
pTGFB1*	TGFB1	transforming growth factor beta 1	2.32
AJ814326	B4GALT1	UDP-Gal:betaGlcNAc beta 1,4- galactosyltransferase polypeptide 1	2.45
**DEATH**			
**EC24T0 > SA24T0**			
CN821828	AGT	angiotensinogen	3.73
BG693016	BTG1	B-cell translocation gene 1, anti-proliferative	2.67
BF076579	BAX	BCL2-associated X protein	2.02
AJ818096	CEBPG	CCAAT/enhancer binding protein (C/EBP), gamma	2.19
CO888526	EMP1	epithelial membrane protein 1	2.30
CO892245	GADD45G	growth arrest and DNA-damage-inducible, gamma	2.91
AJ818246	HSPD1	heat shock 60 kDa protein 1	2.34
AM037100	NUPR1	nuclear protein 1	2.27
CO883017	PHLPP	PH domain and leucine rich repeat protein phosphatise	2.26
CO885391	TAX1BP1	Tax1 (human T-cell leukemia virus type I) binding protein 1	3.05
AM025615	TRIB3	tribbles homolog 3	3.02
**SA24T0 > EC24T0**			
CN822971	CALR	calreticulin	2.36
BF604066	CD74	CD74 molecule, major histocompatibility complex, class II invariant chain	2.43
pCSF2*	CSF2	colony stimulating factor 2 (granulocyte-macrophage)	2.57
CN824202	GSK3B	glycogen synthase kinase 3 beta	2.02
pTGFB1*	TGFB1	transforming growth factor beta 1	2.32
AJ814326	B4GALT1	UDP-Gal:betaGlcNAc beta 1,4- galactosyltransferase polypeptide 1	2.45
**CELL ACTIVATION**			
**EC24T0 > SA24T0**			
CN821828	AGT	angiotensinogen	3.73
BF076579	BAX	BCL2-associated X protein	2.02
AJ818096	CEBPG	CCAAT/enhancer binding protein (C/EBP), gamma	2.19
**SA24T0 > EC24T0**			
BF604066	CD74	CD74 molecule, major histocompatibility complex, class II invariant chain	2.43
pCSF2*	CSF2	colony stimulating factor 2 (granulocyte-macrophage)	2.57
AJ692384	GLMN	glomulin, FKBP associated protein	2.01
CO876411	THY1	Thy-1 cell surface antigen	2.10
pTGFB1*	TGFB1	transforming growth factor beta 1	2.32

A single accession number is listed for genes represented by multiple clones. Average fold differences were calculated when multiple clones represented a transcript. * pTGFB1 and pCSF2 are amplicons generated specifically for the Bov20K cDNA microarray.

Closer analysis of the gene list revealed that several genes previously associated with the mammary gland response to bacterial infection are differentially expressed between SA24T0 and EC24T0. These include signal transducer and activator of transcription 3 (STAT3), LBP, CXCL2, BCL2-associated X protein (BAX), B-cell translocation gene 1 (BTG1), metallothioneins and LCN2, which are all expressed at higher levels in EC24T0 samples. This suggests that the EC24T0 samples are responding to infection of neighbouring udder quarters and this accounts for the majority of the transcriptional differences observed between the control quarters.

**Figure 2:**
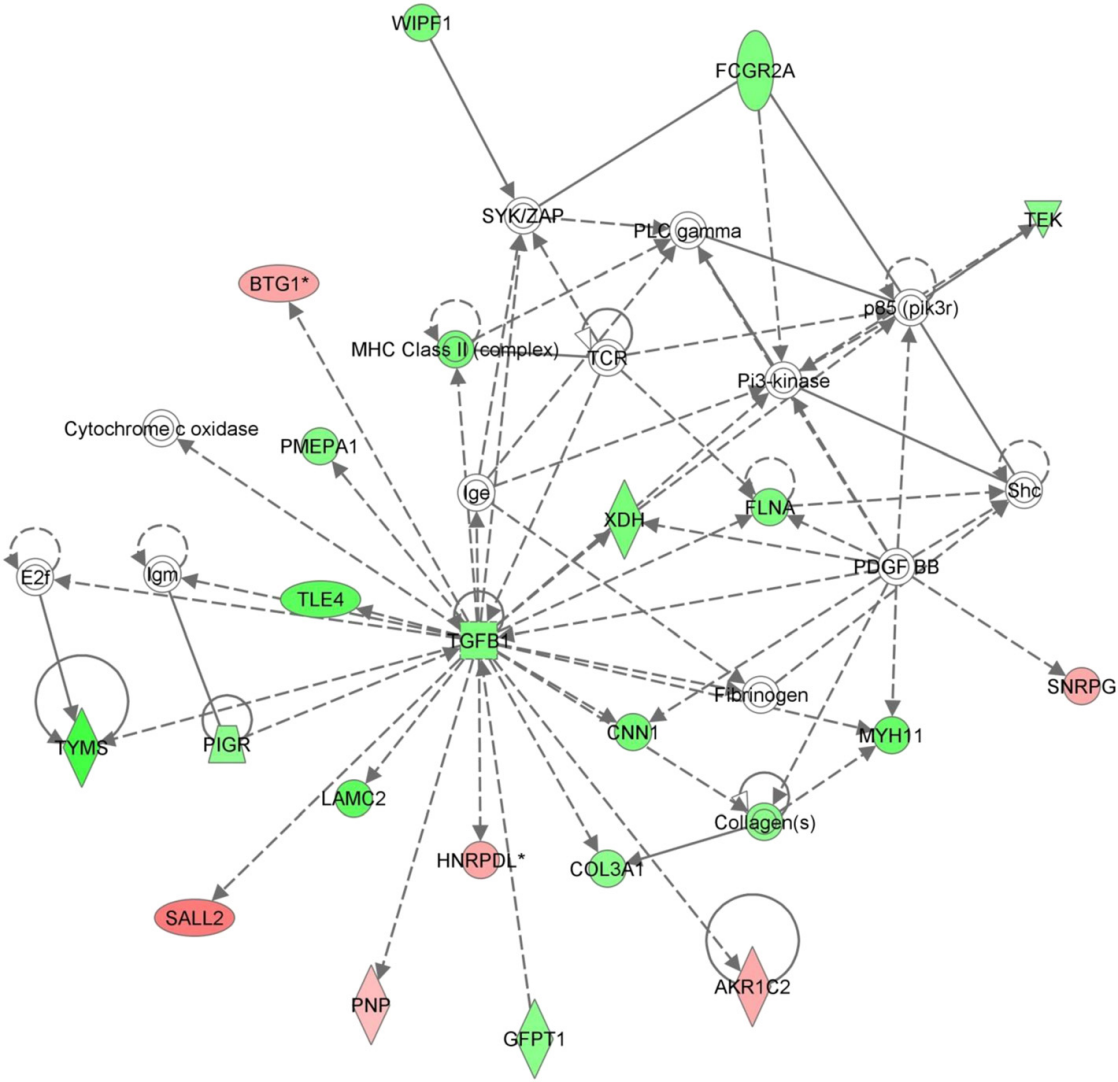
**TGFB1 Nework. **Ingenuity pathway analysis of the EC24T0 v SA24T0 DEG list identified an interaction network involving TGFB1 and associated molecules with a network score of 38. Green denotes molecules that were expressed at higher levels in SA24T0 samples and red denotes those more highly expressed in EC24T0 samples.

The DEG were further analyzed using the Ingenuity Pathway Analysis (IPA) software (V7.0, Ingenuity Systems) as described previously [21]. The most striking functional network found to be over-represented in the DEG list involved transforming growth factor beta 1 (TGFB1) and associated molecules (Figure 2). Of the 23 genes in this network that were in the DEG list, 17 were expressed at higher levels in SA24T0 samples than EC24T0 samples. It is unclear if this is due to up-regulation of these genes in SA24T0 or down-regulation in EC24T0, but the involvement of TGFB1 in immunosuppression suggests that regulation of these genes may have a profound effect on the ability of the mammary gland quarter to respond to infection.

### Validation of microarray results comparing EC24T0 and SA24T0

**Figure 3:**
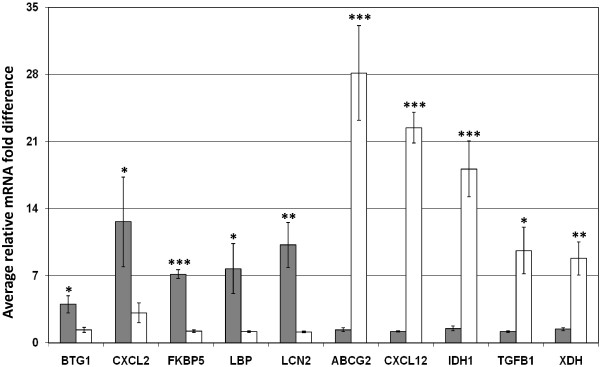
**Validation of EC24T0 v SA24T0 differentially expressed genes. **Summary of the quantitative RT-PCR (RT-qPCR) results for ten selected genes that were identified from the microarray data analysis as exhibiting differential expression between the control quarters from *E. coli *(EC24T0) and *S. aureus *(SA24T0) infected cattle. The results are represented as the average mRNA level detected in EC24T0 (grey bars) and SA24T0 (white bars) relative to the sample with the lowest expression. The bars denote standard error of the mean, *, ** and *** denote that the expression difference is statistically significant with *P* < 0.05, *P* < 0.005 and *P* < 0.0005 respectively.

Ten genes that exhibited differential expression between EC24T0 and SA24T0 were chosen for validation studies by quantitative RT-PCR (RT-qPCR). Analysis of the microarray results suggested that five of these genes; BTG1, CXCL2, FKBP5, LBP and LCN2, were expressed at higher levels in EC24T0 samples. The RT-qPCR results for all five genes were statistically significant and agreed with the microarray results (Figure 3). The other five genes; ATP-binding cassette, sub-family G (WHITE), member 2 (ABCG2), CXCL12, isocitrate dehydrogenase 1 (NADP+), soluble (IDH1), TGFB1 and xanthine dehydrogenase (XDH), were chosen from those that exhibited greater expression in SA24T0 than EC24T0 samples. The RT-qPCR analysis revealed that all five are expressed at statistically significantly higher levels in SA24T0 than EC24T0 samples (Figure 3), including ABCG2 which was expressed on average 20.9 fold higher levels in SA24T0 than EC24T0 samples.

### Genes exhibiting differential expression in control quarters from animals infected with *S. aureus* for 24 and 72 hours

To confirm that the variation between udder quarters was due only to events occurring in EC24T0, the transcriptome of SA24T0 was compared with that of the control quarters from the 72 hours *S. aureus* infection (SA72T0). No clones exhibited differential expression using a *FDR* less than 0.05. However, using less stringent statistical analyses revealed 115 clones that exhibited differential expression (*P* value ≤ 0.01, fold difference ≥ 2), which are listed in Additional file 2. Eighty one of these clones are annotated, representing 76 genes. Twenty two annotated genes (24 clones) were expressed at significantly higher levels in SA72T0. The remaining 54 annotated genes (57 clones), over two thirds of the differentially expressed genes, were expressed at significantly higher levels in SA24T0. The clone exhibiting the highest expression level compared to SA72T0 represents the cell surface marker CD4, with 4.5 fold higher expression. This suggests that the cellular composition of the control udder quarters differed depending on the time of infection of neighbouring quarters. SOD2, which was identified in the earlier analysis as an up-regulated gene during *S. aureus* infection, was expressed 2.3 fold higher in SA24T0 than SA72T0. Similarly, other genes associated with the mammary gland response to infection are expressed at higher levels in SA24T0, e.g. S100A8, S100A12, CXCL10, interleukin (IL) 1B and lactotransferrin (LTF).

**Figure 4:**
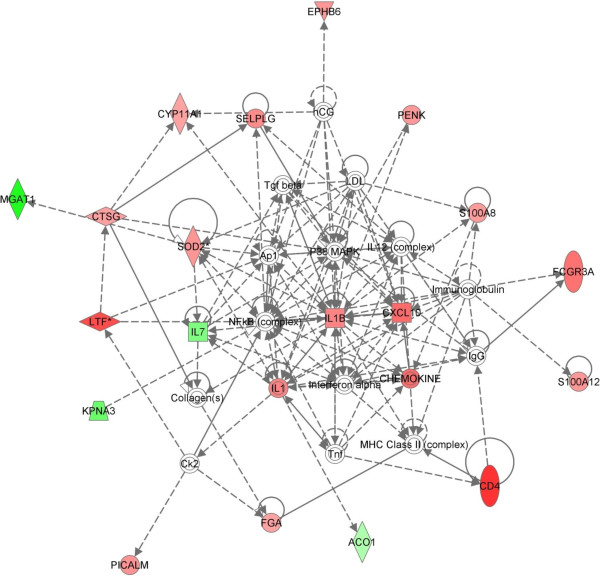
**IL1 Network. **Ingenuity pathway analysis of the SA24T0 v SA72T0 DEG list identified an interaction network involving the pro-inflammatory cytokine IL1 with a network score of 42. Red denotes molecules that were expressed at higher levels in SA24T0 samples and green denotes those more highly expressed in SA72T0 samples.

Gene enrichment analysis using DAVID revealed that several GO Biological Process terms are over-represented in the DEG list (Table 3). The GO term exhibiting the greatest enrichment was Response to Stress. Other over-represented GO terms include; Cell Proliferation, Cell Development, Immune Response and Defence Response. The majority of genes associated with these GO terms are expressed at higher levels in SA24T0 samples. Furthermore, subsequent IPA analysis revealed that the DEG list contained 21 genes that are involved in the functional network with the pro-inflammatory cytokine IL1 at its hub and 17 of these were expressed at higher levels in SA24T0 than SA72T0 samples (Figure 4). These results suggest that uninfected quarters respond to the infection of neighbouring quarters with *S. aureus* for at least 24 hours, but this response had waned by 72 hours post infection. Therefore, of the three sets of control samples generated in this study the transcriptome of SA72T0 samples most closely resembles that of an uninfected quarter. This explains the low number of DEG identified in the initial analysis of the transcriptional response of infected quarters to infection (Table 1). The EC24T0 and SA24T0 samples were used as the baseline to identify DEG, but the response of these quarters to the infection of neighbouring quarters was sufficient to mask the transcriptional response of the infected quarters.

**Table 3:** **Major gene ontology biological process classifications represented by genes differentially expressed between control quarters from cows infected with *S. aureus *for 24 hours (SA24T0) and 72 hours (SA72T0)**

**Clone accession no.**	**Gene symbol**	**gene**	**Fold Difference**
**RESPONSE TO STRESS**			
**SA24T0 > SA72T0**			
CO891789	ASF1A	ASF1 anti-silencing function 1 homolog A	2.53
pCXCL10*	CXCL10	chemokine (C-X-C motif) ligand 10	3.28
AJ693004	CYP11A1	cytochrome P450, family 11, subfamily A, polypeptide 1	2.03
AM018876	FGA	fibrinogen alpha chain	2.03
pIL1B*	IL1B	interleukin 1, beta	2.72
AM037229	PENK	proenkephalin	2.23
BM256666	S100A8	S100 calcium binding protein A8 (calgranulin A)	2.19
BF655078	S100A12	S100 calcium binding protein A12 (calgranulin C)	2.29
AM031467	SOD2	superoxide dismutase 2, mitochondrial	2.30
CO875497	TP53BP1	tumor protein p53 binding protein 1	2.68
AM022673	XRCC2	X-ray repair complementing defective repair in Chinese hamster cells 2	3.22
**SA72T0 > SA24T0**			
CK847668	BRCA2	breast cancer 2, early onset	3.47
**CELL PROLIFERATION**			
**SA24T0 > SA72T0**			
AM009066	ACSL6	acyl-CoA synthetase long-chain family member 6	3.27
pCXCL10*	CXCL10	chemokine (C-X-C motif) ligand 10	3.28
CO874867	CSRP2	cysteine and glycine-rich protein 2	2.26
AM018876	FGA	fibrinogen alpha chain	2.03
pIL1B*	IL1B	interleukin 1, beta	2.72
AM024609	STIL	SCL/TAL1 interrupting locus	2.69
**SA72T0 > SA24T0**			
CK847668	BRCA2	breast cancer 2, early onset	3.47
BI680983	INHA	inhibin, alpha	2.63
AJ815547	IL7	interleukin 7	3.38
**CELL DEVELOPMENT**			
**SA24T0 > SA72T0**			
AM009066	ACSL6	acyl-CoA synthetase long-chain family member 6	3.27
CO874867	CSRP2	cysteine and glycine-rich protein 2	2.26
BM256725	GZMH	granzyme H	2.35
pIL1B*	IL1B	interleukin 1, beta	2.72
CO887046	LRRC4C	leucine rich repeat containing 4C	2.46
AM031467	SOD2	superoxide dismutase 2, mitochondrial	2.30
CO892784	ZEB2	zinc finger E-box binding homeobox 2	2.84
**SA72T0 > SA24T0**			
AJ817391	AFF4	AF4/FMR2 family, member 4	3.20
CK847668	BRCA2	breast cancer 2, early onset	3.47
BI680983	INHA	inhibin, alpha	2.63
AJ815547	IL7	interleukin 7	3.38
**IMMUNE RESPONSE**			
**SA24T0 > SA72T0**			
AM011262	CTSG	cathepsin G	2.09
BF706666	CD4	CD4 molecule	4.50
pCXCL10*	CXCL10	chemokine (C-X-C motif) ligand 10	3.28
pFCGR3A*	FCGR3A	Fc fragment of IgG, low affinity IIIa, receptor (CD16)	3.09
AM023116	GBP4	guanylate binding protein 4	2.16
pIL1B*	IL1B	interleukin 1, beta	2.72
AM011258	LTF	lactotransferrin	3.48
**SA72T0 > SA24T0**			
AJ815547	IL7	interleukin 7	3.38
**DEFENSE RESPONSE**			
**SA24T0 > SA72T0**			
pCXCL10*	CXCL10	chemokine (C-X-C motif) ligand 10	3.28
pIL1B*	IL1B	interleukin 1, beta	2.72
AM011258	LTF	lactotransferrin	3.48
AM037229	PENK	proenkephalin	2.23
BM256666	S100A8	S100 calcium binding protein A8 (calgranulin A)	2.19
BF655078	S100A12	S100 calcium binding protein A12 (calgranulin C)	2.29

A single accession number is listed for genes represented by multiple clones. Average fold differences were calculated for superoxide dismutase 2, mitochondrial and lactotransferrin, which are represented by multiple clones. * pCXCL10, pFCGR3A and pIL1B are amplicons generated specifically for the Bov20K cDNA microarray.

### Validation of microarray results comparing SA24T0 and SA72T0

**Figure 5:**
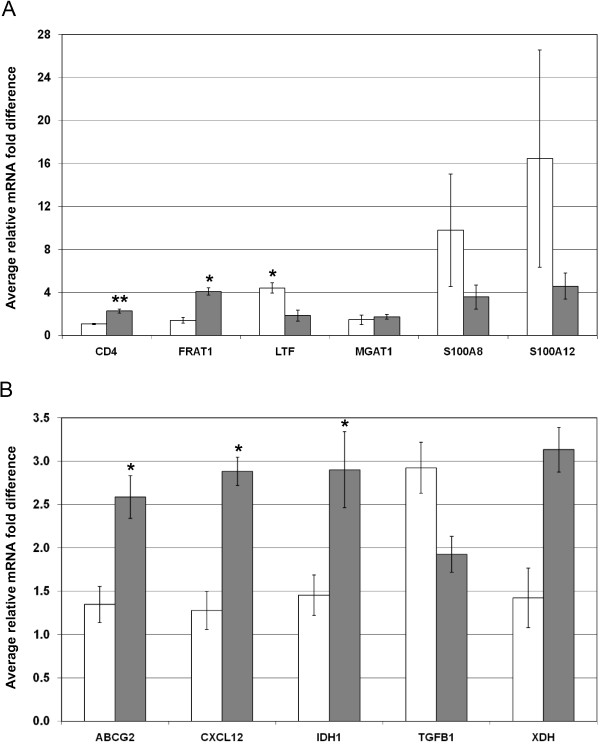
**Validation of SA24T0 v SA72T0 differentially expressed genes. **Summary of the quantitative RT-PCR (RT-qPCR) results for selected genes between the control quarters of animals infected with *S. aureus *for 24 hours (SA24T0) or 72 hours (SA72T0). The results are represented as the average mRNA level detected in SA24T0 (white bars) and SA72T0 (grey bars) relative to the sample with the lowest expression. The bars denote standard error of the mean, * and ** denote that the expression difference is statistically significant with *P* < 0.05 and *P* < 0.005 respectively. A. RT-qPCR results for six selected genes that were identified from the microarray data analysis as exhibiting differential expression between SA24T0 and SA72T0. B. RT-qPCR results comparing the differential expression of ABCG2, CXCL12, IDH1, TGFB1 and XDH between SA24T0 and SA72T0 samples.

The validity of the DEG identified from the comparison of SA24T0 and SA72T0 using less stringent statistical analysis was investigated by RT-qPCR analysis of the expression levels of six genes in the RNA samples. Analysis of the microarray results suggested that CD4 was the most highly expressed gene in SA24T0 compared to SA72T0. However, the RT-qPCR revealed the opposite pattern of expression, with 2.1 fold higher levels in SA72T0 samples (*P* < 0.005) (Figure 5A). Furthermore, mannosyl (alpha-1,3-)-glycoprotein beta-1,2-N-acetylglucosaminyltransferase (MGAT1), which according to the microarray analysis was the most highly expressed gene in SA72T0 compared to SA24T0, exhibited no differential expression by RT-qPCR analysis (Figure 5A). However, frequently rearranged in advanced T-cell lymphomas (FRAT1) and LTF exhibited the same pattern of expression by RT-qPCR and microarray analyses. FRAT1 was expressed at on average 2.9 fold higher levels in SA72T0 samples (*P* < 0.05), whilst LTF was expressed at on average 2.4 fold higher levels in SA24T0 samples (*P* < 0.05) (Figure 5A). Furthermore, although S100A8 and S100A12 did not exhibit statistically significant differential expression by RT-qPCR analysis, the results followed the same trend as observed with the microarray results, with 2.7 fold and 3.6 fold higher expression in SA24T0 samples respectively (Figure 5A). The disparity between the microarray and RT-qPCR analyses may relate to the use of different RNA samples from the same udder quarters suggesting that there are localized differences in the response to infection of neighbouring quarters. Alternatively, the disparity may be due to the small number of out-bred animals used in this study.

In addition, the gene expression of the ten genes used to validate the microarray results for the comparison of EC24T0 and SA24T0 were also measured in the SA72T0 samples. No significant difference in expression was observed in the five genes more highly expressed in EC24T0 samples than SA24T0 samples (data not shown). However, four of the five genes more highly expressed in SA24T0 than EC24T0 samples were found to be more highly expressed in SA72T0 than SA24T0 samples and three showed statistical significance (Figure 5B). None of these four genes were identified from the microarray analysis comparison of SA24T0 and SA72T0. Levels of ABCG2, CXCL12 and IDH1 were all statistically significantly higher in SA72T0 than SA24T0 samples, with on average 1.9, 2.3 and 2.0 fold greater expression. Although not statistically significant XDH was on average 2.2 fold higher in SA72T0 compared to SA24T0 samples (*P* = 0.053). In contrast, TGFB1 levels were higher in SA24T0 samples compared to SA72T0 samples, albeit not to a statistically significant level. Therefore, although not in complete agreement with the microarray data, the RT-qPCR results confirm that the transcriptional profiles of uninfected mammary gland quarters differ during the course of *S. aureus* infection of neighbouring quarters.

## Discussion

Studies involving farm animals require the use of a large number of animals to obtain statistically meaningful data because these out-bred populations exhibit considerable genetic variation. Therefore, it is appealing to use intra-animal controls where possible, since this reduces genetic variation and fewer biological replicates are required. The quarters of the bovine mammary gland have been considered as separate physiological entities and therefore the use of one or more quarters as controls is a generally accepted experimental design [3-7,11-16]. However, the results reported here demonstrate that the mammary gland quarters are interdependent during infection, with a profound change in the transcriptome of one quarter when neighbouring quarters are infected with *S. aureus* and especially *E. coli*. The control quarters remained bacteriologically negative throughout the study period [10] and therefore the observed differences are not due to contamination. The transcriptional response of the uninfected quarters was sufficiently high that it resulted in the masking of the response in infected quarters, when they were used as the base-line for DEG analysis (Table 1). In contrast, previous studies investigating the bovine mammary gland response to infection which have used within-animal controls have identified large numbers of differentially expressed genes [4-7]. However, in the earlier studies the animals were only challenged once, whilst in this study the animals were inoculated three times over 24 hours, which may have resulted in a greater signal triggering the response in the uninfected quarters.

Although a direct comparison between uninfected quarters from healthy and uninfected animals was not made in this study, the effect of infection in neighbouring quarters can be inferred from the differences observed between the various control samples. Recently, a microarray experiment comparing the transcriptome of uninfected quarters from healthy and *E. coli* infected animals was reported [17]. A total of 476 DEG were identified, confirming that *E. coli* infection alters the transcriptional response of neighbouring mammary gland quarters. Over twenty eight percent (53) of the DEG identified in our comparison of *E. coli* and *S. aureus* control quarters were also found in this study, which utilized the bovine Affymetrix platform, including ABCG2 and FKBP5. The direction of the transcriptional change observed in these 53 genes was the same in both studies, i.e. those up-regulated during *E. coli* infection of neighbouring quarters [17] were found at higher levels in EC24T0 samples than SA24T0 samples, suggesting that the differential expression of at least these 53 genes is associated with *E. coli* infection and not *S. aureus* infection. The broader intensity plots of the EC24T0 samples illustrated that there was a greater range of transcriptional change in these samples compared to the reference than observed in the SAT0 samples. This may suggest that the vast majority of the differential expression is due to the *E. coli* infection of neighbouring quarters. Furthermore, the transcriptome of the uninfected quarters was indistinguishable from that of quarters infected with *E. coli* for six hours.

However, the design of the experiment reported here does not allow us to conclusively say that the transcriptional differences are all due to changes in expression in the *E. coli* control quarters. Indeed, the comparison of control quarters from animals infected with *S. aureus* for 24 and 72 hours has revealed that *S. aureus* also induces transcriptional changes in neighbouring quarters 24 hours post infection, which may account for some of the differences observed between SA24T0 and EC24T0. Furthermore, XDH, which is involved in the generation of the bactericidal agent peroxynitrate and is believed to play an important role in the mammary gland immune response (reviewed by [22]), is expressed at significantly higher levels in SA24T0 than EC24T0 and therefore may be induced during *S. aureus* infection of neighbouring quarters. Moreover, additional evidence that *S. aureus* infection induces a response in neighbouring quarters has recently been reported. The expression of the acute phase proteins serum amyloid A3 (SAA3) and haptoglobin (HP) as well as the β-defensin DEFB5 was elevated in uninfected control quarters from animals infected with a low virulence strain of *S. aureus* compared to tissue from healthy animals [23].

The initial statistical analysis of the microarray data comparing the *S. aureus* control quarters from the different time courses failed to detect any significantly differentially expressed genes. Although this could be a genuine result, *S. aureus* infection generally induces a milder immune response than *E. coli*, modulating gene expression to a lesser extent at the site of infection, which would impact on the signals being detected by the neighbouring quarters. This variation, compounded with the well-known fact that individual cattle differ greatly in their response to udder infections (reviewed by [24]), would result in considerable variability in the response of neighbouring quarters to *S. aureus* infection and could account for the lack of statistical significance, which would be further exacerbated by the low number of biological replicates included in this study. Indeed, lowering the stringency of the microarray analysis allowed the identification of over 70 genes, including several genes known to be associated with the mammary gland response to infection, e.g. SOD2, IL1B, LTF, S100A8 and S100A12. Nearly one sixth of the differentially expressed genes are associated with the response of cells to stress. Furthermore, IPA revealed that a network of IL1 and associated molecules were expressed at higher levels in SA24T0 samples than in the SA72T0 samples, which are a well-known trigger for inflammation. Paradoxically, the cattle infected with *S. aureus* did not show signs of inflammation, even in the infused udder quarters, 24 hours post infection [10], when the elevated levels of IL1 and related molecules were detected in this study. Conversely, at 72 hours, when the IL1 response was lower in uninfected quarters, the infected quarters showed up-regulation of inflammation-associated genes [10]. Therefore, *S. aureus* infection may initially elicit a slight systemic inflammatory response that induces IL1 expression in uninfected quarters, which disappears during sustained infection. It is unclear if *S. aureus* actively contributes to the down-regulation of the systemic response, which may contribute to the typically sub-clinical disease pattern observed in *S. aureus* mastitis.

Some of the transcriptional changes may relate to changes in the cellular composition of the mammary gland. Bacterial infections induce the influx of immune cells, predominantly neutrophils, into the infected mammary gland quarter, which leads to increasing SCC in milk. The SCC measured in the control quarters from animals included in this study increased during the time course of both *E. coli* and *S. aureus* infection [10]. This is in agreement with previous studies which found significantly higher SCC in milk from uninfected quarters of naturally infected animals compared to those from healthy animals [25-27]. Moreover, the percentage of neutrophils was significantly higher, whilst the percentage of macrophages was significantly lower, in milk from uninfected quarters from animals with mastitis compared to those from healthy animals [27]. Differential expression of cell surface markers, e.g. CD74, Fc receptors and CD4, are indicative of differences in the cellular composition of the control quarters from *E. coli* and *S. aureus* infected animals. Levels of CD4^+^ T lymphocytes increase in milk during *S. aureus* infection [26,28] and the results reported here suggest that some cells may originate from the uninfected quarters.

The influx of immune cells may not account for all the transcriptional changes of uninfected quarters. The response of the first quarter to infection with *E. coli* was significantly different from that of the second infected quarter after the same period of infection. The profound increase in SCC observed in milk from the first quarter 12 hours post infection was not observed in milk samples from the second quarter infected 12 hours later [10]. The earlier infection appears to have primed the neighbouring mammary gland quarters to respond differently to infection. Similar results have been reported recently in an experimental infection study where one animal showed signs of infection in one udder quarter before the experiment started [7]. This animal was still included in the study, using the other, uninfected udder quarters. The proinflammatory cytokine response, i.e. up-regulation of IL1B, IL6, IL8 and tumour necrosis factor (TNF), of this animal was more pronounced than that observed in other animals included in the study [7]. The priming of the mammary gland may persist after infection. Experimental *E. coli* infection of individual quarters fourteen days after infection of neighbouring quarters resulted in significantly less bacteria being isolated from the milk than observed with the initial infection [29]. Furthermore, it was recently reported that priming udders with a low dose of *E. coli* lipopolysaccharide (LPS) protects against experimental infection with *E. coli* pathogens [30]. Moreover, LPS priming induces sustained expression of bactericidal factors (e.g. β-defensins) by bovine mammary epithelial cells (MEC), whilst reducing the over expression of pro-inflammatory cytokines which may be harmful to the animal [31]. However, there is little overlap in DEG from primed MEC and those observed in the uninfected quarters included in this study, suggesting that different mechanisms may be involved in priming neighbouring quarters.

TGFB1 levels were observed to be much lower in the control quarters from *E. coli* infected animals than those from animals infected with *S. aureus*. Therefore, it is interesting to hypothesize that the priming of the udder quarters involves the suppression of levels of TGFB1 and associated molecules which was revealed by IPA. Moreover, the same pattern of TGFB3 expression has previously been reported using the same experimental samples [32]. The TGFBs are pleiotropic cytokines which play an important role in multiple biological processes including regulating the adaptive and innate immune responses. TGFB1 inhibits inflammation by, amongst other mechanisms, suppressing the activation of several immune cells, e.g. NK cells and macrophages, and although chemotactic, can inhibit neutrophil transmigration (reviewed by [33]). Further analysis has shown that significantly lower levels of TGFB1 mRNA is present in the uninfected quarters of *E. coli* infected animals included in this study than in the quarters of healthy animals involved in another study [17] (Additional file 3). It appears that animals down regulate the expression of TGFB1 in the udder when a neighbouring quarter has been challenged with *E. coli* pathogens. This reduction in the level of the immunosuppressor TGFB1 would lower the threshold for triggering the induction of a variety of immunologically relevant genes, resulting in a faster and more robust response to infection. In contrast, the weaker stimulus elicited by invading *S. aureus* pathogens maintains, or possibly enhances (Additional file 3), the relatively high levels of TGFB1 present in uninfected quarters, thereby buffering immune stimuli in the healthy, uninfected udder quarter.

Response to Stress was one of the major GO Biological Processes categories identified in both the comparisons of EC24T0 and SA24T0 and that of *S. aureus* control quarters. Many of the genes within this category have previously been identified as being involved in the immune response to mastitis, including LBP, STAT3, S100A8 and SOD2. However, many of the most differentially regulated genes, e.g. ABCG2 and FKBP5, have not, to our knowledge, previously been associated with mastitis. Both ABCG2 and FKBP5 were also identified as DEG in the recent transcriptional comparison of uninfected quarters from healthy and mastitic animals [17], suggesting that their differential expression was due to the infection of neighbouring quarters with *E. coli*. Furthermore, the expression of both genes was modulated in *E. coli* infected mammary gland quarters [17].

ABCG2, also known as breast cancer resistance gene (BCRP), is a xenobiotic transporter that is expressed at high levels in lactating mammary glands of cows, humans and mice [34,35]. ABCG2 plays an important role in the transport of nutrients into milk, especially riboflavin (vitamin B_2_) [36] and has been identified as the candidate gene underlying a quantitative trait locus on bovine chromosome 6 that affects milk yield and composition [37,38]. Therefore, the down-regulation of ABCG2 in quarters neighbouring *E. coli* infected quarters may be associated with the decrease in milk yield and casein synthesis, which was observed in these animals [10,39] and may imply a nutrient deficiency in milk from these animals.

The immunophilin FKBP5 has several functions, including mediating the action of the immunosuppressive drug FK506. The FKBP5-FK506 complex binds protein phosphatase 3, catalytic subunit, alpha isozyme (PPP3CA), also known as calcineurin, thus preventing it from phosphorylating and activating NFAT transcription factors, which in turn prevents T cell activation and proliferation [40]. FKBP5 is highly expressed in murine T cells [40] and therefore the up-regulation observed during *E. coli* infection may be due to lymphocyte migration into mammary gland tissue. In addition, FKBP5 is induced by glucocorticoids and is believed to be involved in the negative feedback loop for glucocorticoid receptor signalling [41]. FKBP5, along with heat shock protein 90 (HSP90), is a component of the glucocortcoid receptor complex. The HSP90 family member HSP90AB1 exhibits a similar expression profile as FKBP5 in both this study and Mitterheumer et al. [17]. Glucocorticoids are able to modulate NF-κB activity by a FKBP5-dependent pathway [42]. FKBP5 interacts with conserved helix-loop-helix ubiquitous kinase (IKKα), which phosphorylates nuclear factor of kappa light polypeptide gene enhancer in B-cells inhibitor, alpha (IκBα), the inhibitor of NF-κB, resulting in IκBα proteosome-mediated degradation and the nuclear translocation and activation of NF-κB [43].

The mechanism by which the infection of neighbouring quarters affects the transcriptome of uninfected quarters is unknown. Mastitis induces a systemic effect, inducing transcriptional changes in the liver [44], including the production of acute phase proteins which are released into the blood that circulates around all the udder quarters. This has been postulated to explain the transcriptional profile in healthy quarters of animals infected with *E. coli*[17]. Furthermore, it has become clear that endogenous damage or danger signals [45] also lead to inflammation [46] and evidence now supports their role in the response to pathogens [47]. Therefore, it is possible that the uninfected udder quarters are responding to such danger or damage signals released into the extracellular environment following infection in the neighbouring quarters. However, analysis of TLR and β-defensin expression in the local and peripheral lymph nodes of animals included in this study revealed that a response was only observed in the lymph nodes draining the quarters infected with *E. coli* for 12 and 24 hours [10], suggesting that the systemic effect is quite limited. Local cross-talk between the udder quarters must, therefore, be responsible for priming the neighbouring udder quarters. Indeed, lymphocytes have been shown to be capable of migrating between mammary gland quarters [48] and therefore the presence of a more localized interaction between quarters is probable and requires further investigation.

## Conclusions

It has previously been shown that during *E. coli* infection the immune status of neighbouring, sterile quarters is modified [17]. The results presented here provide further evidence of this and also illustrate that a similar phenomenon occurs during *S. aureus* infection. However, the details of the transcriptional response induced by these pathogens appear to be quite distinct, but are difficult to separate out fully in this study due to the experimental design used. However, the data suggest that the induced transcriptional changes prime the uninfected quarters to prevent or limit the spread of infection. The outcome of mastitis infections is influenced by multiple factors, including the virulence of the pathogen and host genetics. It is clear that the previous exposure of animals to pathogens is also important. Considering the frequency of mastitic infections on dairy farms the pathogen primed state may be the predominant status of uninfected quarters and therefore worthy of further investigation. Furthermore, the results described here illustrate that the four quarters of the bovine mammary gland cannot be considered as separate experimental units during infection studies. This has important ramifications for the design of experimental challenge studies, as the use of inappropriate controls can result in the masking of transcriptional changes during infection. Therefore caution should be taken when using within-animal controls.

## Methods

### Experimental design of the infection study

The design of the infection study has been described in detail previously in accordance with ARRIVE guidelines [10]. This work was carried out under the approval of the ethics committee of the regional government in Hannover, Germany (No. 509.6-42502-03**/**678). Briefly, twelve Holstein cows in the middle of their first lactation were challenged over 24 or 72 hours with 500 cfu *E. coli* strain 1303 or 10,000 cfu *S. aureus* strain 1027. The animals were randomly divided into three groups. Four animals were inoculated intramammary with *E. coli* in the front right, hind right and hind left udder quarters at 0 (T24), 12 (T12) and 18 (T6) hours after the trial started respectively. The front left quarter received 2 ml sterile saline at the start of the experiment. The animals were euthanized after 24 hours, with a penetrating captive bolt gun followed by exsanguination, and tissue samples were collected asceptically from each udder quarter. The second group of four animals were inoculated with *S. aureus* over the same 24 hour period. The third group of four animals were inoculated with *S. aureus* in both right quarters (T72) and inoculated with 2 ml sterile saline in the front left quarter at 0 hours. The hind left quarter was inoculated at 60 hours (T12) after the start of the experiment. The cows were euthanized after 72 hours as described above and tissue samples were collected from each udder quarter. The control quarters from animals in these three groups are referred to as EC24T0, SA24T0 and SA72T0 respectively in this paper. All animals infected with *E. coli* developed acute mastitis within 12 hours, although the major clinical symptoms, e.g. udder swelling and elevated SCC, where only observed in the inoculated mammary gland quarters [10]. In contrast, clinical signs of mastitis were only observed in animals infected with *S. aureus* for 72 hours [10].

### RNA samples

A 5 cm^3^ tissue sample were removed asceptically from the inner area of each udder quarter, 10 cm dorsal of the milk cistern, within 5–10 minutes of culling. A small section of the centre of this sample (1.0 x 0.5 x 0.5 cm) was stored in RNAlater (Ambion) for RNA isolation. Total RNA was extracted as described previously [49] by caesium chloride gradient followed by phenol/chloroform extraction and sodium acetate precipitation. One hundred micrograms of each RNA sample was purified further using RNeasy mini columns (Qiagen) using the manufacturer’s protocols. The quality and quantity of the resulting RNA was determined by gel electrophoresis and Agilent 2100 Bioanalyzer.

### Microarray experiment

#### Experimental design

The RNA samples from each mammary gland quarter were analyzed separately and hybridized in competition with a pooled reference sample, made up of all the RNA samples. A common reference design was used to allow the multifactorial analysis of the complete experiment. The reference sample was labelled with cyanine (Cy) 3 and the treatment sample with Cy5 on each microarray slide. The microarray experiment therefore consisted of a total of 48 microarray slides. The microarray data has been submitted to ArrayExpress and assigned the accession number E-TABM-704.

#### RNA labelling & hybridizations

Fluorescently labelled cDNA was generated from 20 μg total RNA using the FairPlay II microarray labelling kit (Stratagene), following the manufacturer’s protocol. The labelled cDNA was purified using a DyeEx spin column (Qiagen) and the labelling efficiency was determined by running 0.5 μl of each sample on a 1% agarose gel. Labelled cDNA was hybridized onto the ARK-Genomics *Bos taurus* 20 K v1.0 microarray (ArrayExpress accession no. A-MEXP-1402). Hybridizations were carried out in a GeneTac automated hybridization station (Genomic Solutions) to control for reactions conditions and washing of the arrays. The Cy3 and Cy5 labelled cDNA were mixed and added to 125 μl hybridization solution (ARK-Genomics protocols: http://www.ark-genomics.org) and hybridized onto the microarray for 12 hours. The microarray slides were then washed in wash buffers of increasing stringency (Genomic Solutions). After removal from the hybridization station the microarray slides were sequentially washed in post-wash buffer and isopropanol for one minute before being dried by centrifugation at 220 *g* for six minutes. The dried slides were scanned in a Scanarray 5000 XL scanner (GSI Lumonics) at constant laser power of 75% and 73% for Cy3 and Cy5 respectively.

#### Data analysis

Microarray spot intensity and quality data were extracted from the scanned images using the BlueFuse software version II (BlueGnome). Preliminary analysis was carried out using the GeneSpring software package (Agilent). Further analysis was carried out using modifications of the Limma package of Bioconductor [50], with additional plotting from the Marray package. The microarray data exhibited low signal to noise ratios, which resulted in excessive variability at low intensity values. To reduce this each channel was augmented by 256. The normalization and analysis steps were weighted using the BlueFuse quality measure, confidence values between 0 and 1 [51], and manually excluded spots were given the minimum confidence value. Each slide was normalized separately, using the log_2_-ratios of treatment to reference intensities for all the non-control spots. The data was normalized using a 2-step process of spatial followed by intensity dependent bias correction for each print-tip, as described previously [52]. The spatial bias was corrected by subtracting corresponding row and column means from each spot [53]. The intensity dependent bias was removed by block-lowess [54].

Genes that were differentially expressed between the infected and control samples were identified by analyzing the means of replicate spots. The Limma eBayes correction [55] was used to shrink the residual variances of each gene towards their median value. Comparisons between the infected and control samples were assessed by 1-way ANOVA followed by *t-*tests. *FDR* values for each comparison were calculated using the method of Benjamini and Hochberg [56]. Genes with *FDR* values less than or equal to 0.05 were considered as significantly different. To refine the list of differentially expressed genes further to those of probable biological relevance, only those genes exhibiting a 2 fold or greater average difference were considered.

### Validation of microarray experiments

RT-qPCR analysis was used to validate the microarray results for sixteen differentially expressed genes. Oligonucleotides were designed for each gene using Primer3 [57] and Netprimer (Biosoft International) software (Table 4). Total RNA was isolated from a different sample of mammary gland tissue than that used for the microarray experiment. The tissue was powdered under liquid nitrogen and mixed with the chaotropic RLT lysis buffer (Qiagen). RNA was extracted with Phenol (Ambion) and purified using RNeasy spin columns (Qiagen) including a DNase digestion step (Qiagen) according to the manufacturer’s instructions. The RNA quality was assessed by agarose gel electrophoresis and NanoDrop ND-1000 spectrophotometer (Thermo Scientific).

**Table 4:** Details of the oligonucleotides used for the quantitative RT-PCR analysis

**Gene**	**Gene Symbol**	**Direction**	**Sequence (5′-3′)**	**Product size**
ATP-binding cassette, sub-family G	ABCG2	F	CCTTCGGCTTCCAACAACT	129
(WHITE), member 2		R	CCAGACACACCACGGATAAA	
B-cell translocation gene 1, anti-	BTG1	F	TGAAAGTAGCAAGTGACCAGAA	192
proliferative		R	CAAGGAGAGTTACAAACCAGACC	
CD4 molecule	CD4	F	GGCAGAACCACAGACTCACA	133
		R	GACAAACAAGCCCAAAGGAA	
Chemokine (C-X-C motif) ligand 2	CXCL2	F	GCCAAACCGAAGTCATAGCC	213
		R	TGGAAACCAGCCATTCTCTTC	
Chemokine (C-X-C motif) ligand 12	CXCL12	F	TTGAAAGCCTGACCCATAAA	146
		R	GACAGTGGCAGCAGAGAAG	
FK506 binding protein 5	FKBP5	F	CCACAGCAGCATCACACAC	189
		R	GGGAAGGCTAATCCAGAACC	
Frequently rearranged in advanced T-	FRAT1	F	GCCCAAAGGACAAGGATG	186
cell lymphomas		R	CCAAGAACAAGCACCTCAAA	
Isocitrate dehydrogenase 1 (NADP+),	IDH1	F	CTCTCAAGGGTAAAGGCAAA	113
soluble		R	TTCACAAAGGTGGCATAACTG	
Lactotransferrin	LTF	F	CTGTGGCTAAATTCTTCTCTGC	187
		R	TTAACAAAAGCCACGTCTCCAG	
Lipocalin 2	LCN2	F	CCAACTACGAGCTGAAGGAAGAC	103
		R	TGGGAGCTTGGGACAAAAGT	
Lipopolysaccharide binding protein	LBP	F	AGGGCAAGGTGAAAGACAGG	178
		R	TGGAGTCAGAGAGGGTGTGG	
Mannosyl (alpha-1,3-)-glycoprotein beta-	MGAT1	F	TCTCCATCCAGTCCTTTCCA	191
1,2-N-acetylglucosaminyltransferase		R	ACATTGCTCTCCAACCCATC	
S100 calcium binding protein A8	S100A8	F	TCTATTTTGGGGAGACCTGGTG	203
		R	CCAAGTGTCCGCATCCTTTT	
S100 calcium binding protein A12	S100A12	F	AGGGAATCATCAACATCTTCCAC	172
		R	TCTTTATCGGCATCCAGGTCTT	
Transforming growth factor, beta 1	TGFB1	F	AATGGTGGAATACGGCAACA	121
		R	CCGAGAGAGCAACACAGGTTC	
Xanthine dehydrogenase	XDH	F	TCAGGATGATGGTTGGAAGA	193
		R	GGGAGTTAGGACATAGCACGA	
Chromosome alignment maintaining	CHAMP1	F	AGCAGTGACCAAGAGCAGGT	205
phosphoprotein 1		R	TCATAGCACGACAGCAACAA	

F and R denote forward and reverse primers respectively.

The RT-qPCRs for twelve genes were carried out as described previously [58]. The relative quantities of mRNA were calculated using the method described by Pfaffl [59]. The RT-qPCR results for chromosome alignment maintaining phosphoprotein 1 (CHAMP1) were used to calculate differences in the template RNA levels and thereby standardize the results for the genes of interest. The RT-qPCR for the remaining four genes; chemokine (C-X-C motif) ligand (CXCL) 2, lipocalin 2 (LCN2), S100 calcium bind proteins (S100) A8 and S100A12, were carried out as described previously [60] and relative copy numbers were calculated. These results were converted to fold differences to standardize the results for all sixteen genes. All samples were measured twice from two independent cDNA preparations. The quality and authenticity of the resulting RT-qPCR products were assessed by agarose gel electrophoresis and sequencing. The relative quantity values were transformed on the log_2_ scale before statistical analyses to stabilize the variance and make them comparable to the log (intensity ratios) from the microarray analysis. The effect of infection in neighbouring quarters was examined by *t*-test analysis (Genstat 8.1, Lawes Agricultural Trust, Rothamsted).

## Competing interests

The authors declare that they have no competing interests.

## Authors’ contributions

KJ designed the microarray experiment, analyzed the differentially expressed genes and drafted the manuscript. RT constructed the microarray and oversaw the microarray experiments. KJ and JG carried out the RT-qPCR analysis. JG analyzed the gene lists using Ingenuity. HMS, WP, HZ and HJS designed and carried out the infection study and collected the samples. HMS conceived the study and coordinated the use of the infection samples in the microarray experiment. EJG was involved in the microarray design and coordination of the study. All authors read and approved the final manuscript.

## Supplementary Material

Additional file 1 **List of clones exhibiting statistically significant differential expression between control quarters from *E. coli *and *S. aureus *infected cows. **An excel file listing the 255 clones, representing 187 known genes, exhibiting differential expression between control quarters from *E. coli* and *S. aureus *infected cows (fold difference ≥ 2, *FDR *< 0.05). The fold differences between the control quarters from *E. coli *(EC24T0) and *S. aureus *(SA24T0) infected cattle are displayed as a positive number when EC24T0>SA24T0 (highlighted in orange) and a negative number when SA24T0>EC24T0 (highlighted in green). Genes shown in bold are represented by more than one clone. The calculated *FDR *values are included. Click here for file

Additional file 2 **List of clones exhibiting statistically significant differential expression between control quarters from cows infected with *S. aureus *for 24 and 72 hours. **An excel file listing the 115 clones, representing 76 known genes, exhibiting differential expression between control quarters from cows infected with *S. aureus *for 24 (SA24T0) and 72 hours (SA72T0) (fold difference ≥ 2, *P *< 0.01). The fold differences between the control quarters from animals infected with *S. aureus *for 24 hours (SA24T0) or 72 hours (SA72T0) are displayed as a positive number when SA24T0>SA72T0 (highlighted in orange) and a negative number when SA72T0>SA24T0 (highlighted in green). Genes shown in bold are represented by more than one clone. The calculated *P *values are included. Click here for file

Additional file 3 **Infection of neighbouring quarters affects the levels of TGFB1 mRNA in uninfected quarters. **Comparison of TGFB1 mRNA levels in uninfected quarters of cattle infected for 24 hours with *S. aureus *(SA24T0), *E. coli *(EC24T0) or from healthy, uninfected (HEALTHY) cattle. TGFB1 levels are expressed as relative copy numbers and the bars denote standard error of the mean. The statistical significance is indicated as P values and n.s. denotes not significant. The HEALTHY samples were included in a previously published study [17]. Animals in both studies were selected using the same criteria and hormonally synchronized using the same procedure. Click here for file

## References

[B1] Viguier C Arora S Gilmartin N Welbeck K O’Kennedy R Mastitis detection: current trends and future perspectives Trends Biotech 2009 27 486 493 10.1016/j.tibtech.2009.05.004 19616330

[B2] Schukken YH Günther J Fitzpatrick J Fontaine MC Goetze L Holst O Leigh J Petzl W Schuberth HJ Sipka A Smith DGE Quesnell R Watts J Yancey R Zerbe H Gurjar A Zadoks RN Seyfert HM Host-response patterns of intramammary infections in dairy cows Vet Immunol Immunopath 2011 144 270 289 10.1016/j.vetimm.2011.08.022 21955443

[B3] Rinaldi M Li RW Bannerman DD Daniels KM Evock-Clover C Silva MVB Paape MJ Van Ryssen B Burvenich C Capuco AV A sentinel function for teat tissues in dairy cows: dominant innate immune response elements define early response to E. coli mastitis Funct Integr Genomics 2010 10 21 38 19727872 10.1007/s10142-009-0133-z

[B4] Buitenhuis B Røntved CM Edwards SM Ingvartsen KL Sørensen P In depth analysis of genes and pathways of the mammary gland involved in the pathogenesis of bovine Escherichia coli-mastitis BMC Genomics 2011 12 130 21352611 10.1186/1471-2164-12-130 PMC3053262

[B5] Moyes KM Drackley JK Morin DE Bionaz M Rodriguez-Zas SL Everts RE Lewin HA Loor JJ Gene network and pathway analysis of bovine mammary tissue challenged with Streptococcus uberis reveals induction of cell proliferation and inhibition of PPARγ signalling as potential mechanism for the negative relationships between immune response and lipid metabolism BMC Genomics 2009 10 542 19925655 10.1186/1471-2164-10-542 PMC2784807

[B6] Swanson KM Stelwagen K Dobson J Henderson HV Davis SR Farr VC Singh K Transcriptome profiling of Streptococcus uberis-induced mastitis reveals fundamental differences between immune gene expression in the mammary gland and in a primary cell culture model J Dairy Sci 2009 92 117 129 19109270 10.3168/jds.2008-1382

[B7] Lutzow YCS Donaldson L Gray CP Vuocolo T Pearson RD Reverter A Byrne KA Sheehy PA Windon R Tellam RL Identification of immune genes and proteins involved in the response of bovine mammary tissue to Staphylococcus aureus infection BMC Vet Res 2008 4 18 18513449 10.1186/1746-6148-4-18 PMC2430192

[B8] Genini S Badaoui B Sclep G Bishop SC Waddington D van der Pinard LMH Klopp C Cabau C Seyfert HM Petzl W Jensen K Glass EJ de Greeff A Smith HE Smits MA Olsaker I Boman GM Pisoni G Moroni P Castiglioni B Cremonesi P Del Corvo M Foulon E Foucras G Rupp R Giuffra E Strengthening insights into host reponses to mastitis infection in ruminants by combining heterogeneous microarray data sources BMC Genomics 2011 12 225 21569310 10.1186/1471-2164-12-225 PMC3118214

[B9] Loor JJ Moyes KM Bionaz M Functional adaptations of the transcriptome to mastitis-causing pathogens: the mammary gland and beyond J Mammary Gland Biol Neoplasia 2011 16 305 322 21968536 10.1007/s10911-011-9232-2

[B10] Petzl W Zerbe H Günther J Yang W Seyfert H-M Nürnberg G Schuberth HJ Escherichia coli, but not Staphylococcus aureus, triggers an early increased expression of factors contributing to the innate immune defense in the udder of the cow Vet Res 2008 39 18 18258172 10.1051/vetres:2007057

[B11] Chang CC Winter AJ Norcross NL Immune response in the bovine mammary gland after intestinal, local and systemic immunization Infect Immun 1981 31 650 659 7012016 10.1128/iai.31.2.650-659.1981 PMC351359

[B12] Mehrzad J Dosogne H Meyer E Burvenich C Local and systemic effects of endotoxin mastitis on the chemiluminescence of milk and blood neutrophils in dairy cows Vet Res 2001 32 131 144 11361149 10.1051/vetres:2001100

[B13] Bannerman DD Paape MJ Lee JW Zhao X Hope JC Rainard P Escherichia coli and Staphylococcus aureus elicit differential innate immune responses following intramammary infection Clin Diagn Lab Immunol 2004 11 463 472 15138171 10.1128/CDLI.11.3.463-472.2004 PMC404560

[B14] Schmitz S Pfaffl MW Meyer HHD Bruckmaier RM Short-term changes of mRNA expression of various inflammatory factors and milk proteins in mammary tissue during LPS-induced mastitis Domest Anim Endocrin 2004 26 111 126 10.1016/j.domaniend.2003.09.003 14757184

[B15] Silva LFP Liesman JS Etchbarne BE Nielsen MSW VandeHaar MJ Intramammary infusion of IGF-1 increases bromodeoxyuridine labelling in mammary epithelial cells of prepubertal heifers J Dairy Sci 2005 88 2771 2773 16027190 10.3168/jds.s0022-0302(05)72956-8

[B16] Grönlund U Johannisson A Waller KP Changes in blood and milk lymphocyte sub-populations during acute and chronic phases of Staphylococcus aureus induced bovine mastitis Res Vet Sci 2006 80 147 154 15982680 10.1016/j.rvsc.2005.05.002

[B17] Mitterhuemer S Petzl W Krebs S Mehne D Klanner A Wolf E Zerbe H Blum H Escherichia coli infection induces distinct local and systemic transcriptome responses in the mammary gland BMC Genomics 2010 11 138 20184744 10.1186/1471-2164-11-138 PMC2846913

[B18] Donaldson L Vuocolo T Gray C Strandberg Y Reverter A McWilliam S Wang Y Byrne K Tellam R Construction and validation of a bovine innate immune microarray BMC Genomics 2005 6 135 16176586 10.1186/1471-2164-6-135 PMC1261263

[B19] Dennis G Sherman BT Hosack DA Yang J Gao W Lane HC Lempicki RA DAVID: database for annotation, visualization and integrated discovery Genome Biol 2003 4 P3 12734009 PMC193660

[B20] Huang DW Sherman BT Lempicki RA Systematic and integrative analysis of large gene lists using DAVID bioinformatics resources Nat Protoc 2009 4 44 57 19131956 10.1038/nprot.2008.211

[B21] Günther J Esch K Poschadel N Petzl W Zerbe H Mitterhuemer S Blum H Seyfert HM Comparative kinetics of Escherichia coli- and Staphylococcus aureus-specific activation of key immune pathways in mammary epithelial cells demonstrates that S. aureus elicits a delayed response dominated by interleukin-6 (IL-6) but not IL-1A or tumor necrosis factor alpha Infect Immun 2011 79 695 707 21115717 10.1128/IAI.01071-10 PMC3028868

[B22] Rainard P Riollet C Innate immunity of the bovine mammary gland Vet Res 2006 37 369 400 16611554 10.1051/vetres:2006007

[B23] Whelehan CJ Meade KG Eckersall PD Young FJ O’Farrelly CO Experimental Staphylococcus aureus infection of the mammary gland induces region-specific changes in innate immune gene expression Vet Immunol Immunopathol 2011 140 181 189 21292330 10.1016/j.vetimm.2010.11.013

[B24] Burvenich C Van Merris V Mehrzad J Diez-Fraile A Duchateau L Severity of E. coli mastitis is mainly determined by cow factors Vet Res 2003 34 521 564 14556694 10.1051/vetres:2003023

[B25] Bansal BK Hamann J Grabowski NT Singh KB Variation in the composition of selected milk fraction samples from healthy and mastitic quarters and its significance for mastitis diagnosis J Dairy Res 2005 72 144 152 15909679 10.1017/s0022029905000798

[B26] Hamann J Schröder A Merle R Hogeveen H Differential cell count and interdependence of udder quarters Mastitis in dairy production: current knowledge and future solutions 2005 The Netherlands: Wageningen Academic Publishers 190 195

[B27] Merle R Schröder A Hamann J Cell function in the bovine mammary gland: a preliminary study on interdependence of healthy and infected udder quarters J Dairy Res 2007 74 174 179 17227597 10.1017/S002202990600238X

[B28] Riollet C Rainard P Poutrel B Cell subpopulations and cytokine expression in cow milk in response to chronic Staphylococcus aureus infection J Dairy Sci 2001 84 1077 1084 11384034 10.3168/jds.S0022-0302(01)74568-7

[B29] Suojala L Orro T Järvinen H Saatsi J Pyörälä S Acute phase response in two consecutive experimentally induced E. coli intramammary infections in dairy cows Acta Vet Scand 2008 50 18 18554387 10.1186/1751-0147-50-18 PMC2440372

[B30] Petzl W Günther J Pfister T Sauter-Louis C Goetze L von Aulock S Hafner-Marx A Schuberth H-J Seyfert H-M Zerbe H Lipopolysaccharide pretreatment of the udder protects against experimental Escherichia coli mastitis Innate Immun 2012 18 467 477 21990573 10.1177/1753425911422407

[B31] Günther J Petzl W Zerbe H Schuberth H-J Koczan D Goetze L Seyfert H-M Lipopolysaccharide priming enhances expression of effectors of immune defence while decreasing expression of pro-inflammatory cytokines in mammary epithelia cells from cows BMC Genomics 2012 13 17 22235868 10.1186/1471-2164-13-17 PMC3315725

[B32] Yang W Zerbe H Petzl W Brunner RM Gunther J Draing C von Aulock S Schuberth HJ Seyfert HM Bovine TLR2 and TLR4 properly transduce signals from Staphylococcus aureus and E. coli, but S. aureus fails to both activate NF-κB in mammary epithelial cells and to quickly induce TNFα and interleukin-8 (CXCL8) expression in the udder Mol Immunol 2008 45 1385 1397 17936907 10.1016/j.molimm.2007.09.004

[B33] Li MO Wan YY Sanjabi S Robertson AKL Flavell RA Transforming growth factor-β regulation of immune responses Annu Rev Immunol 2006 24 99 146 16551245 10.1146/annurev.immunol.24.021605.090737

[B34] Jonker JW Merino G Musters S van Herwaarden AE Bolscher E Wagenaar E Mesman E Dale TC Schinkel AH The breast cancer resistance protein BCRP (ABCG2) concentrates drugs and carcinogenic xenotoxins into milk Nat Med 2005 11 127 129 15685169 10.1038/nm1186

[B35] Mani O Sorensen MT Sejrsen K Bruckmaier RM Albrecht C Differential expression and localization of lipid transporters in the bovine mammary gland during the pregnancy-lactation cycle J Dairy Sci 2009 92 3744 3756 19620656 10.3168/jds.2009-2063

[B36] van Herwaarden AE Wagenaar E Merino G Jonker JW Rosing H Beijnen JH Schinkel AH Multidrug transporter ABCG2/breast cancer resistance protein secretes riboflavin (vitamin B2) into milk Mol Cell Biol 2007 27 1247 1253 17145775 10.1128/MCB.01621-06 PMC1800714

[B37] Cohen-Zinder M Seroussi E Larkin DM Loor JJ Loor JJ van der Everts WA Lee J-H Drackley JK Band MR Hernandez AG Shani M Lewin HA Weller JI Ron M Identification of a missense mutation in the bovine ABCG2 gene with a major effect on the QTL on chromosome 6 affecting milk yield and composition in Holstein cattle Genome Res 2005 15 936 944 15998908 10.1101/gr.3806705 PMC1172037

[B38] Weikard R Widmann P Buitkamp J Emmerling R Kuehn C Revisiting the quantitative trait loci for milk production traits on BTA6 Anim Genet 2011 43 318 323 22486504 10.1111/j.1365-2052.2011.02258.x

[B39] Vanselow J Yang W Herrmann J Zerbe H Schuberth H-J Petzl W Tomek W Seyfert H-M DNA-remethylation around a STAT5-binding enhancer in the αS1-casein promoter is associated with abrupt shutdown of αS1-casein synthesis during acute mastitis J Mol Endocrinol 2006 37 463 477 17170087 10.1677/jme.1.02131

[B40] Baughman G Wiederrecht GJ Campbell NF Martin MM Bourgeois S FKBP51, a novel T-cell-specific immunophilin capable of calcineurin inhibition Mol Cell Biol 1995 15 4395 4402 7542743 10.1128/mcb.15.8.4395 PMC230679

[B41] Denny WB Valentine DL Reynolds PD Smith DF Scammell JG Squirrel monkey immunophilin FKBP51 is a potent inhibitor of glucocorticoid receptor binding Endocrinology 2000 141 4107 4113 11089542 10.1210/endo.141.11.7785

[B42] Park J Kim M Na G Jeon I Kwon Y Kim J Youn H Koo Y Glucocorticoids modulate NF-κB-dependent gene expression by up-regulating FKBP51 expression in Newcastle disease virus-infected chickens Mol Cell Endo 2007 278 7 17 10.1016/j.mce.2007.08.002 17870233

[B43] Bouwmeester T Bauch A Ruffner H Angrand P-O Bergamini G Croughton K Cruciat C Eberhard D Gagneur J Ghidelli S Hopf C Huhse B Mangano R Michon AM Schirle M Schleg J Schwab M Stein MA Bauer A Casari G Drewes G Gavin AC Jackson DB Joberty G Naubauer G Rick J Kuster B Superti-Furga G A physical and functional map of the human TNF-α/NF-κB signal transduction pathway Nat Cell Biol 2004 6 97 105 14743216 10.1038/ncb1086

[B44] Jiang L Sørensen P Røntved C Vels L Ingvartsen KL Gene expression profiling of liver from dairy cows treated intra-mammary with lipopolysaccharide BMC Genomics 2008 9 443 18816405 10.1186/1471-2164-9-443 PMC2576255

[B45] Matzinger P Tolerance, danger, and the extended family Annu Rev Immunol 1994 12 991 1045 8011301 10.1146/annurev.iy.12.040194.005015

[B46] Manson J Thiemermann C Brohi K Trauma alarmins as activators of damage-induced inflammation Brit J Surg 2012 99 Suppl 1 12 20 22441851 10.1002/bjs.7717

[B47] Fontana MF Vance RE Two signal models in innate immunity Immunol Rev 2011 243 26 39 21884165 10.1111/j.1600-065X.2011.01037.x

[B48] Kimura K Harp JA Goff JP Olsen SC Lymphocytes from one side of the bovine mammary gland migrate to the contra lateral gland and lymph node tissue Vet Immunol Immunopathol 2005 108 409 415 16111770 10.1016/j.vetimm.2005.05.014

[B49] Chirgwin JM Przybyla AE MacDonald RJ Rutter WJ Isolation of biologically active ribonucleic acid from sources enriched in ribonuclease Biochemistry 1979 18 5294 5299 518835 10.1021/bi00591a005

[B50] Smyth GK Gentleman R, Carey V, Dudoit S, Irizarry R, Huber W Bioinformatics and Computational Biology Solutions using R and Bioconductor Bioinformatics and Computational Biology Solutions using R and Bioconductor 2005 New York: Springer 397 420

[B51] Jaffrézic F de Koning DJ Boettcher PJ Bonnet A Buitenhuis B Closset R Déjean A Delmas C Detilleux JC Dovč P Duval M Foulley J-L Hedegaard J Hornshøj H Hulsegge I Janss L Jensen K Jiang L Lavrič M Lê Cao K-A Lund MS Malinverni R Marot G Nie H Petzl W Pool MH Robert-Granié C San Cristobal M van Schothorst EM Schuberth H-J Analysis of the real EADGENE dataset: comparison of methods and guidelines for data normalisation and selection of differentially expressed genes Genet Sel Evol 2007 39 633 650 18053573 10.1186/1297-9686-39-6-633 PMC2682811

[B52] Jensen K Talbot R Paxton E Waddington D Glass EJ Development and validation of a bovine macrophage specific cDNA microarray BMC Genomics 2006 7 224 16948847 10.1186/1471-2164-7-224 PMC1590031

[B53] Baird D Johnstone P Wilson T Normalization of microarray data using a spatial mixed model analysis which includes splines Bioinformatics 2004 20 3196 3205 15231532 10.1093/bioinformatics/bth384

[B54] Smyth GK Speed TP Normalization of cDNA microarray data Methods 2003 31 265 273 14597310 10.1016/s1046-2023(03)00155-5

[B55] Smyth GK Linear models and empirical Bayes methods for assessing differential expression in microarray experiments Stat Appl Genet Mol Biol 2004 3 3 10.2202/1544-6115.1027 16646809

[B56] Benjamini Y Hochberg Y Controlling the false discovery rate: a practical and powerful approach to multiple testing J Roy Stat Soc B 1995 57 289 300

[B57] Rozen S Skaletsky HJ Krawetz S, Misener S Primer3 on the WWW for general users and for biologist programmers Bioinformatics Methods and Protocols: Methods in Molecular Biology 2000 Totowa: Humana Press 365 386 10.1385/1-59259-192-2:365 10547847

[B58] Jensen K Paxton E Waddington D Talbot R Darghouth M Glass EJ Differences in the transcriptional responses induced by Theileria annulata infection in bovine monocytes derived from resistant and susceptible cattle breeds Int J Parasitol 2008 38 313 325 17949724 10.1016/j.ijpara.2007.08.007

[B59] Pfaffl MW A new mathematical model for relative quantification in real-time RT-PCR Nucleic Acids Res 2001 29 e45 11328886 10.1093/nar/29.9.e45 PMC55695

[B60] Goldammer T Zerbe H Molenaar A Schuberth HJ Brunner RM Kata SR Seyfert HM Mastitis increases mammary mRNA abundance of beta-defensin 5, toll-like-receptor 2 (TLR2), and TLR4 but not TLR9 in cattle Clin Diagn Lab Immunol 2004 11 174 185 14715566 10.1128/CDLI.11.1.174-185.2004 PMC321333

