# Supplementary figures and images for: Escherichia coli- and Staphylococcus aureus-induced mastitis differentially modulate transcriptional responses in neighbouring uninfected bovine mammary gland quarters
Source: BMC Genomics. 2013 Jan 16;14:36. doi: 10.1186/1471-2164-14-36 (PMC3598231; doi:10.1186/1471-2164-14-36)

## Slide 1
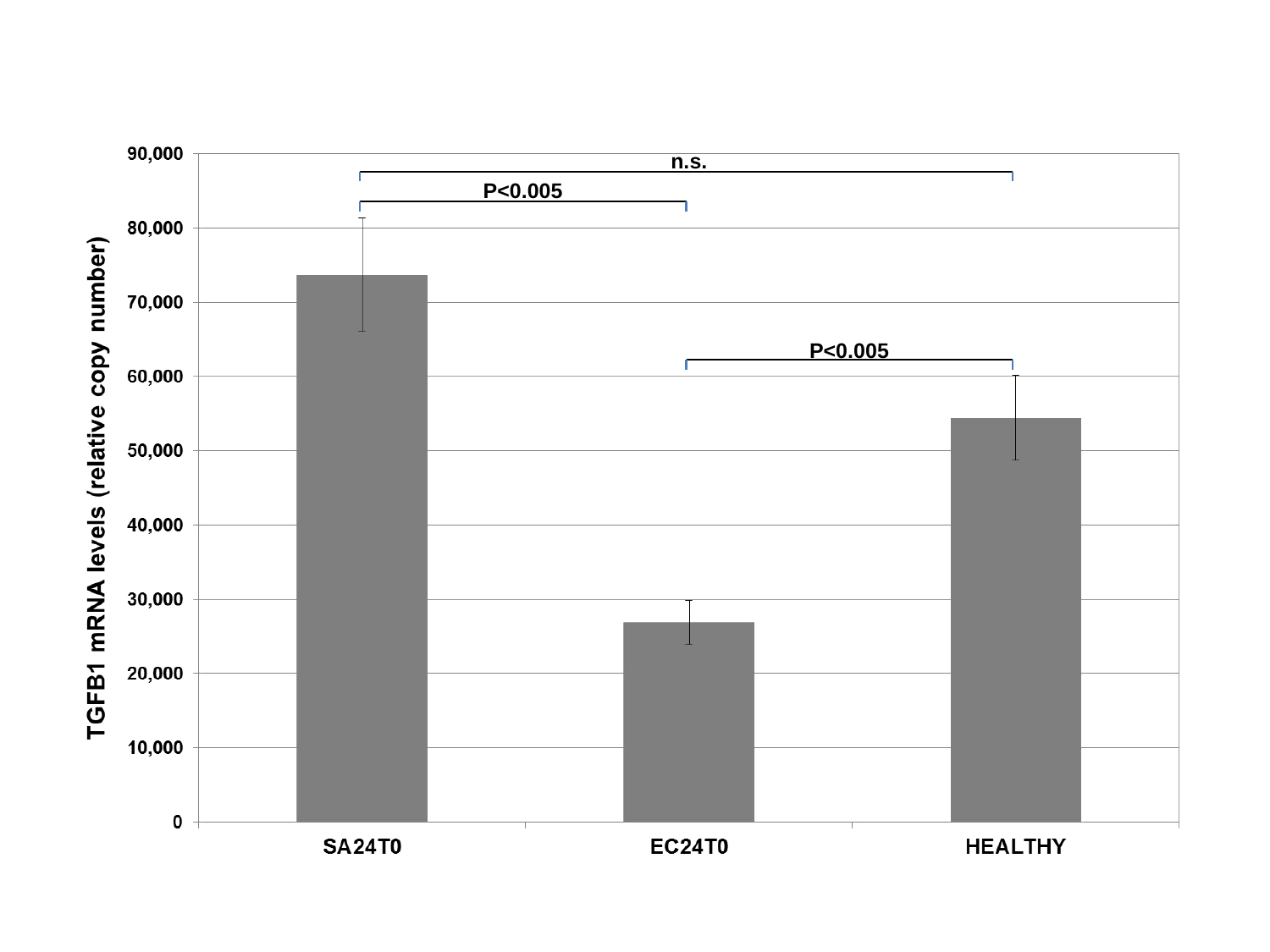

n.s.
P<0.005
P<0.005

Supplement: Additional file 3 — Infection of neighbouring quarters affects the levels of TGFB1 mRNA in uninfected quarters. Comparison of TGFB1 mRNA levels in uninfected quarters of cattle infected for 24 hours with S. aureus (SA24T0), E. coli (EC24T0) or from healthy, uninfected (HEALTHY) cattle. TGFB1 levels are expressed as relative copy numbers and the bars denote standard error of the mean. The statistical significance is indicated as P values and n.s. denotes not significant. The HEALTHY samples were included in a previously published study [17]. Animals in both studies were selected using the same criteria and hormonally synchronized using the same procedure. [file 1471-2164-14-36-S3.pptx]
